# Cellulosic/Polyvinyl Alcohol Composite Hydrogel: Synthesis, Characterization and Applications in Tissue Engineering

**DOI:** 10.3390/polym13203598

**Published:** 2021-10-19

**Authors:** Mathilde Stricher, Claude-Olivier Sarde, Erwann Guénin, Christophe Egles, Frédéric Delbecq

**Affiliations:** 1 Université de Technologie de Compiègne, CNRS, Biomechanics and Bioengineering, Centre de Recherche Royallieu, CEDEX CS 60 319, 60 203 Compiègne, France; mathilde.stricher@utc.fr (M.S.); christophe.egles@utc.fr (C.E.); 2 Université de Technologie de Compiègne, ESCOM, TIMR (Integrated Transformations of Renewable Matter), Centre de Recherche Royallieu, CEDEX CS 60 319, 60 203 Compiègne, France; claude-olivier.sarde@utc.fr (C.-O.S.); erwann.guenin@utc.fr (E.G.)

**Keywords:** cellulose dialdehyde, PVA, regeneration process, porous three-dimensional scaffold, biomedical use

## Abstract

The biomedical field still requires composite materials for medical devices and tissue engineering model design. As part of the pursuit of non-animal and non-proteic scaffolds, we propose here a cellulose-based material. In this study, 9%, 18% and 36% dialdehyde-functionalized microcrystalline celluloses (DAC) were synthesized by sodium periodate oxidation. The latter was subsequently coupled to PVA at ratios 1:2, 1:1 and 2:1 by dissolving in N-methyl pyrrolidone and lithium chloride. Moulding and successive rehydration in ethanol and water baths formed soft hydrogels. While oxidation effectiveness was confirmed by dialdehyde content determination for all DAC, we observed increasing hydrolysis associated with particle fragmentation. Imaging, FTIR and XDR analysis highlighted an intertwined DAC/PVA network mainly supported by electrostatic interactions, hemiacetal and acetal linkage. To meet tissue engineering requirements, an interconnected porosity was optimized using 0–50 µm salts. While the role of DAC in strengthening the hydrogel was identified, the oxidation ratio of DAC showed no distinct trend. DAC 9% material exhibited the highest indirect and direct cytocompatibility creating spheroid-like structures. DAC/PVA hydrogels showed physical stability and acceptability *in vivo* that led us to propose our DAC 9%/PVA based material for soft tissue graft application.

## 1. Introduction

Among recent technologies deployed in tissue engineering or regenerative medicine, scaffold creation is a major strategy for the replacement or repair of soft tissues such as skin, nerves, tendons, ligaments, and cartilage. Clinicians need ready-to-use and easy-to-handle devices to replace the traditionally preferred grafts which are, however, limited in terms of availability, compatibility and rejection. The overall goal of a biocompatible scaffold is to promote and drive the cell regenerative response which supports cell signalling toward adhesion, proliferation and extracellular matrix (ECM) deposition while the material degrades in a controllable and non-toxic manner.

Hydrogels are water-swollen macromolecular networks of hydrophilic polymers that can be formed either by physical gelation through variations of temperature, pH, ionic content or freeze-thaw method, or through chemical gelation under covalent cross-linking via chemical conjugation or polymerization generating nanosized pores [1]. Unfortunately, most of the time, these chemical reactions require toxic or harmful reagents such as carbodiimides or dialdehydes (mainly glutaraldehyde). For tissue engineering applications, hydrogels should exhibit high porosity, tissue-like water content, injectability; and tunable permeability, degradability and mechanical property. However, their nanosized mesh limits nutrients and cellular wastes diffusion, drastically inhibiting cell settlement, attachment and proliferation, as well as neo-tissue formation [2]. Thus, additional macro-porosity is often provided through particle leaching, freeze-drying, gas foaming or electrospinning [3].

Natural biomaterials such as proteins or polysaccharides exhibit biocompatibility and degradability due to their resemblance to tissue extracellular matrix [4]. However, they often demonstrate poor mechanical properties. Therefore, combining them with synthetic polymers such as polyvinylpyrrolidone (PVP), polycaprolactone (PCL), poly-ethylene-glycol (PEG), poly-L-lactic acid (PLA), composite hydrogel design tends to enhance their high stretchability and soft tissue-like stiffness [5]. The benefits of composite materials are thus widely recognized and actively investigated [6]. A particular impulse is emerging for the development of cellulosic materials combined with other natural polymers including chitosan, starch, alginates, collagen and hyaluronic acid [7].

Cellulose, the main constituent of higher plants cell walls, is the Earth’s most abundant organic biopolymer. It can be easily extracted as microcrystals, nanocrystals and nanofibrils from rice husk, raw cotton sliver and sisal, as well as from waste paper and agricultural by-products using usual chemical procedures such as acid hydrolysis, chlorination, alkaline extraction, and bleaching [8,9,10,11,12]. Made up of O glycosidic β-(1→4) linked polymer of D-glucose sub-units, cellulose is a hydrophilic fibrous biopolymer featuring both amorphous and crystalline zones held together by Van der Waals forces and hydrogen bonds. The cellulose hydrophobic sheet association and all interchained hydrogen bonding networks stabilize a crystal-like structure limiting its solubility in water and organic solvents that complicates its handling [13]. Recently, some progress was made for dissolving polysaccharides such as starch or cellulose by using ionic liquids or LiCl dissolved in organic solvents such as DMF [14,15]. To date, cellulosic based materials in development take numerous forms: hydrogel films, three-dimensional scaffolds or drug delivery systems (DDS), mainly for their great mechanical strengthening properties and well-known biocompatibility and biodegradability [16,17]. For these purposes, cellulose derivatives could be functionalised via silyl groups, carboxylic acid or aldehydes functions to engage physical and chemical interactions with various other polymers [18,19,20].

Among all chemically modified cellulose, one of the most promising candidates remains the dialdehyde cellulose (DAC) that expresses a reduced number of hydrogen bonds and weakened inter-chains interactions due to the controlled level of oxidation for the glucose subunits. When oxidized, a glucose sub-unit is currently cleaved between two neighbouring carbons holding the −OH functions, this leads to the formation of two distinct aldehyde functions. Thus, the material is expected to become easily soluble in an amide-type solvent such as *N*-methyl pyrrolidone (NMP) or *N*,*N*-dimethylacetamide (DMAc) containing a slight amount of LiCl [21]. For our experiments, we focused exclusively on NMP in regards to its relatively good biological tolerance and its ability to form an organogel when filled with PVA before to the solubilisation of cellulose at an elevated temperature [22].

Polyvinyl alcohol (PVA) is one of the synthetic polymers that can be sustainably produced by the successive conversion of bio-based ethanol to ethylene, forming the vinyl acetate monomers polymerized to produce bio-sourced PVA, followed by subsequent cleavage of the successive acetyl groups [23,24,25]. PVA is a synthetic polymer presenting hydrogel self-assembling abilities under repeated freeze-thaw cycles through physical crosslinking of side hydroxyl groups offering tunable mechanical properties [26]. Its high hydrophilicity without functional groups such as COOH or NH_2_ makes the PVA inherently non-adhesive to proteins but also to cells unable to engage binding surface receptors, favouring cell-cell interactions [27,28]. PVA-coated surfaces have allowed for the formation of reproducible sized spheroids for glioma, dental pulp or hair follicle cells showing excellent cell activity, migration and regenerative potential [29,30,31]. Interestingly, the poor bioactivity of PVA can be balanced through combination with polymers of higher adhesive properties or conjugation with adhesive proteins [32,33,34].

In this work, we aimed at developing and characterizing a cellulose/PVA material modulating DAC oxidation degree and DAC/PVA ratio, for versatile uses as implantable matrices for soft tissue filling or innovative tissue engineering initiatives. Through the meticulous selection of the components (MCC, PVA) and solvents (NMP, ethanol and water), the limitation of the number and duration of reactions, we offer an eco-friendly, economical and easy-to-scale solution for the development of cellulose-based materials.

## 2. Materials and Methods

All chemicals were purchased from ACROS Organics, Carlsbad, CA, USA unless otherwise stated.

### 2.1. Cellulosic Scaffold Fabrication

**Figure 1:**
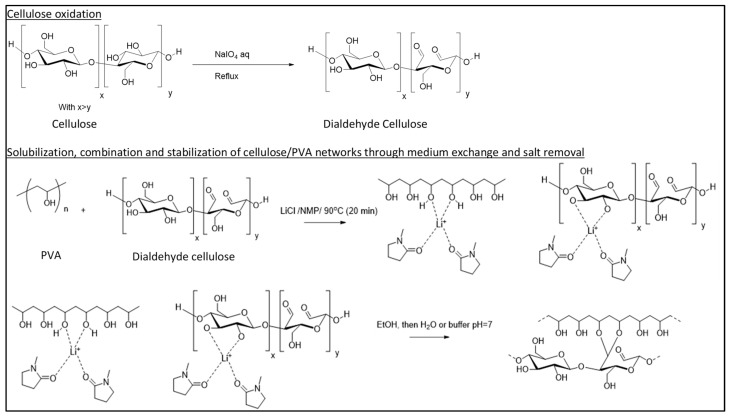
Reaction mechanisms of cellulose dialdehyde oxidation and formulation of cellulosic/PVA scaffolds.

The chemical reactions corresponding to the protocol are shown in Figure 1. Extra pure microcrystalline cellulose (mean particles size: 90 µm) was suspended with 6.25, 12.5 or 25% *w*/*w* sodium periodate in water for 4 h reaction at 90°C under conventional thermal heating with stirring and reflux. Dried DAC powders with theoretical molar dialdehyde content of 9, 18 and 36% were stored at room temperature (RT). Reaction yield is determined from the dried product mass. For the formulation of DAC/PVA (2:1), 3.2 g of DAC and 1.6 g of fully hydrolysed PVA (Sigma Aldrich) were suspended in 20 mL of concentrated 1-Methyl-2-pyrrolidone (NMP; 99.5% Extra Dry) at 12% *w*/*w* Lithium chloride (LiCl; 99%) for 20 min reaction at 90°C under conventional thermal heating with constant stirring. The resulting paste was deposited in pre-made PDMS moulds forming cylindrical samples 16 mm in diameter and 3 mm thick following successive immersion in absolute ethanol (Fisher Bioreagents, Waltham, MA, USA) and ultra-pure water (Millipore, Burlington, MA, USA).

DAC/PVA ratio was also modulated to 1:1 and 1:2. Macro-porosity is promoted by 1 g/mL NaCl (0–500 µm and 50–100 µm; VWR Chemicals; Radnor, PA, USA) addition before casting. Long-term storage of the resulted DAC/PVA scaffolds was ensured in absolute ethanol at 4 °C.

### 2.2. Characterisation of Dialdehyde Celluloses (DACs)

#### 2.2.1. FE-SEM Observations of DACs Morphology

Each set of oxidised cellulose powder was observed using scanning electron microscopy (XL 30-ESEM FEG, Philips, Amsterdam, The Netherlands). The surface of cellulose particles was quantified using Image J software [35].

#### 2.2.2. Quantification of the Aldehyde Content (DC %)

For each DAC sample, the degree of oxidation was estimated following 2 distinct methods: 2,4-dinitrophenylhydrazine (DNPH) and hydroxylamine hydrochloride. For the DNPH method using each sample, 0.1 g of DAC to be tested were dissolved under stirring at room temperature (RT) for 4 h in 30 mL of 16.77 mM DNPH in acidified methanol solution. The reacted yellowed cellulose was filtered, washed with acetone before being dried and weighed. The filtrate was diluted in 400 mL of distilled water. Non-reacted DPNH solution absorbances were measured by UV spectroscopy at 350 nm. The dialdehyde content was thus determined using a calibration curve prepared with DNPH acidified methanol solutions varying from 2.0 to 17 mM. For the hydroxylamine method [36] and each sample, 0.1 g of DAC to be tested were dissolved under stirring at RT for 3 h in 25 mL of 25 mM hydroxylamine hydrochloride (NH_2_OH·HCl) at initial pH of 3.4. Released hydrochloric acid acidifying the solution were then dosed back raising the pH to the initial one via a NaOH solution (0.1 M) under stirring and measurement with a pH meter. The dialdehyde content was thus determined as:

(1) $$DC\left(\%\right)=\frac{V_{\mathrm{NaOH}}\times C_{\mathrm{NaOH}}}{m_{\mathrm{sample}}\times M_{w}}\times \frac{1}{2}\times 100$$

where *M_w_* represents the molar weight of the dialdehyde cellulose unit (160 g·mol^−1^).

### 2.3. Physico-Chemical Characterisations of DAC/PVA Scaffolds

#### 2.3.1. Fourier-Transform Infrared Spectroscopy (FT-IR)

FTIR spectra of dried materials of PVA, 9%, 18% or 36% DAC/PVA (2:1) were recorded with an FT-IR 4000 (Jasco, Tsukuba, Japan) in a range of 400 to 4000 cm^−1^ operated at 4 cm^−1^ resolution using KBr method.

#### 2.3.2. X-ray Diffraction Analysis (XDR) 

XDR w recorded on an X’pert MRD diffractometer (Pan Analytic/Philips, Eindhoven, the Netherlands; 40 Kv, 30 mA) using Cu Kα (λ = 0.151418 nm) radiation. Scans were performed over a 2θ range from 0 to 55, with a step size of 0.018° with a counting time per step of 5 s. The crystallinity index of the cellulosic samples was calculated based on equation (1), where the crystalline *I_Cr_* and the amorphous *I_am_* components are both defined as the peak intensity at 2θ of 22.7° and the minimum intensity around 2θ of 18° [37]:

(2) $$I=\frac{\left(I_{Cr}-I_{am}\right)}{I_{Cr}}\times 100$$

#### 2.3.3. Structure Observations

The microstructure and nanostructure of the DAC/PVA composite scaffolds were evaluated using scanning electron microscopy (XL 30-ESEM FEG, Philips, Amsterdam, The Netherlands) using ethanol dehydrated and dried samples. Rehydrated samples were stained for 15 min with a drop of Calcofluor White Stain and a drop of 10% potassium hydroxide then rinsed in large amounts of water. Cellulose contents of the scaffolds were observed at 433 nm using epifluorescence microscopy (Leica DMI6000 B, Leica Microsystems, Wetzlar, Germany).

#### 2.3.4. Degree of Pore Connectivity, Total Water Content and Swelling Ratio Evaluations

DAC/PVA composites pastilles were weighted at several stages: hydrated ($m_{h}$), dehydrated ($m_{w}$) gently wicked on absorbent paper to remove water entrapped into scaffold cavities and dried ($m_{d}$) under laboratory hood for 24 h. The swelling ratio of the scaffold was recorded via mass measurement ($m_{s}$) upon rehydration into ultrapure water every 15 min for one hour and then regularly until no further mass fluctuations were observed. The degree of pore connectivity (PC), total water content ($Q_{w}$) and Swelling ratio (*α*) were calculated as:

(3) $$PC=\frac{\left(m_{h}-m_{w}\right)}{m_{h}}\times 100$$

(4) $$Q_{W}=\frac{\left(m_{h}-m_{d}\right)}{m_{h}}$$

(5) $$\alpha =\frac{m_{s}}{m_{d}}$$

### 2.4. Mechanical Characterization of Cellulosic Scaffold

#### 2.4.1. Uniaxial Compressive Test

Uniaxial compression tests were conducted on overnight rehydrated cylindrical cellulosic hydrogels (16 mm diameter, 3 mm thick). The wet samples were compressed with a 22 N load cell at a rate of 0.6 mm/min to 35% strain using a Synergie 400 mechanical testing system (MTS system, Eden Prairie, MN, USA). The Young’s modulus was obtained as the slope of the stress-strain curve between 10–20% deformation, avoiding any surface detection bias.

#### 2.4.2. Nanoindentation

Cellulosic hydrogel Young’s moduli were estimated using a PIUMA CHIARO nanoindentation system (Optics11, Amsterdam, The Netherlands). A colloidal probe with a cantilever stiffness of 0.47 N/m and a diameter of 28.5 μm was used. For each hydrogel formulation, three separate samples were tested at five locations by indentation of 15,000 nm at 3 nm/s. Before testing, calibration of the cantilever sensitivity was performed by indenting a hard surface (e.g., Petri dish).

### 2.5. Biological Characterisation of Cellulosic Scaffold

Unless otherwise specified, all reagents were purchased from Gibco, Grand Island, NE, USA. The experiments were performed on DAC/PVA scaffolds (2:1) with 9%, 18% and 36% DAC.

#### 2.5.1. Cytotoxicity Testing

Extracts of sterile materials were formulated following the ISO 109933-5 standards. As a positive control, latex was extracted at 6 cm^2^/mL and test cellulosic materials at 1.25 cm^2^/mL for 24 h at 37 °C on stirring in DMEM medium supplemented with 10% SVF, PenStrep (100 U/mL, 100 µg/mL) and L-glutamine 2 mM. 100 µL of extracts were deposited on L929 mice fibroblast cell monolayers (ATCC-CCL-1, ATCC, Manassas, VA, USA) pre-cultured for 24 h in 96-well plates and incubated for an additional 24 h at 37 °C in the presence of 5% CO2. The medium was then substituted with 120 µL of MTS solution (1:5; Promega, Madison, WI, USA) in a complete medium for 2 h at 37 °C. The absorbance was read at 490 nm. The viability percentage for each material was determined as the absorbance ratio to the untreated control. According to the standards, an extract inducing over 70% viability was considered non-toxic.

#### 2.5.2. HDF Cell Adhesion, Viability, Proliferation and In Vitro Tissue Building Assessments

Neonatal human dermal fibroblasts (HDFn, C-004-5C, Thermo Fisher Scientific, Waltham, MA, USA) were grown in DMEM medium supplemented with 10% FCS (Hyclone, Logan, UT, USA), 2 mM L-glutamine, 100 U/mL penicillin and 100 μg/mL streptomycin at 37 °C with 5% CO_2_. For all experiments, cellulosic materials were seeded with 250,000 HDFn cells. Cell adhesion was examined at 48 h of culture on samples fixed for 15 min with 4% PFA, permeabilized for 5 min with 0.1% Triton X-100, saturated for 10 min with 1% BSA and stained for 30 min with phalloidin/DAPI (1/1000; 1/200) at room temperature in the dark. The nucleus and the actin cytoskeleton were respectively revealed by DAPI and Phalloidin (Ex/Em: 493/517 nm; Ex/Em: 340/488 nm) using confocal microscopy (Zeiss LSM 710, Oberkochen, Germany). Cell viability was also checked at 48 h of culture by 30 min treatment with Fixable Dead Cell Stain, followed by DAPI/Phalloidin staining. To visualise long term cell organisation within the matrix, seeded DAC/PVA scaffolds were maintained in culture for 3 weeks and then stained for DAPI/Phalloidin

#### 2.5.3. Implantation in an Athymic Mice Model

Pathogen-free 5-week-old male athymic mice (Rj: NMRI-Foxn1 nu/nu, JANVIER LABS, Le Genest-Saint-Isle, France; 30 g) were housed in polycarbonate cages, in a temperature and humidity-controlled room, and had free access to food and water *ad libitum*. All the in-vivo experiments were approved by the “Comité Régional d’Ethique en Matière d’Expérimentation Animale de Picardie” (CREMEAP; C2EA-96) and were done in compliance with European directive 2010-EU63. Ethanol sterilized DAC/PVA scaffolds were implanted subcutaneously on the backs of athymic mice. The animals were sacrificed after one month and their skins at implantation point were harvested, observed under a microscope and processed under classical histology procedures (Althisia, Troyes, France). An anatomopathological study of the haematoxylin eosin-stained sections was performed by a veterinary pathologist and ranked as follow (SCIEMPATH BIO, Larcay, Belgium). Local inflammation scores at the implantation site were calculated ranging from 0 (absent) to 4 (severe) following the ISO10993-6 considering the infiltration of cellular inflammation at the interface of the material, the presence of fibrosis/fibrous capsule surrounding the injected filler, necrosis and tissue degeneration. A score of 0 designates that the absence of inflammatory reaction as the cellularity at the interface is similar to the non-injected adjacent dermis. Scores of 1 to 4 were respectively attributed when no inflammatory reaction, low focal to multifocal inflammatory reaction, moderate multifocal to diffuse inflammatory and severe inflammatory reaction showing extensive infiltrates, necrosis or fibrosis were observed at the implant interface.

### 2.6. Statistics

Data are shown as mean ± standard deviations (SD). To determine significant statistical differences, the one-way ANOVA tests and Tukey test were used. Statistical significance was represented as *p*-values < 0.05 (*), *p*-values < 0.01 (**) and *p*-values < 0.001 (***).

## 3. Results

### 3.1. Impact of the Oxidation Level on the DAC Particle Size

**Figure 2:**
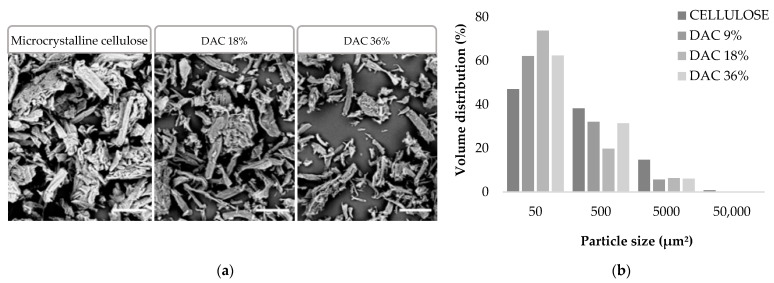
(**a**) MEB visualisation (Scale bar = 100 µm) and (**b**) Granulometric distribution of cellulosic powders.

Extra pure microcrystalline cellulose was oxidised with 6.25, 12.5 or 25% *w*/*w* sodium periodate to obtain three different DAC powders. Yields of 90–95% were obtained for DAC 9% and 18%, while a loss in mass of 40% was observed for DAC 36%. These DAC powders were observed by scanning electron microscopy and quantified by measuring the surface area, the aspects displayed by the cellulose particles show, in Figure 2, a significant fragmentation of the particles compared to the commercial microcrystalline cellulose. After oxidation, a global disappearance of >50,000 µm^2^ particles, a 50% decrease of 5000 to 50,000 µm^2^ particles and a multiplication of 50 to 100 µm^2^ particles going from 47% to 62–74% of total particles were observed. The presence of the generated dialdehyde groups and material crystallinity were also investigated later, respectively, by FTIR and XRD studies when DAC powders were included in the composite hydrogels.

In parallel to the size and shape investigations, the number of aldehyde functions (CHO) was evaluated upon reaction with DNPH in acidic conditions. Indeed, aldehydes were converted to DNP through the formation of hydrazones. The quantification of the unreacted DNPH enabled the determination of the DNPH consumption which gave an aldehyde content estimation in Table 1. The tests were performed in triplicate to ensure the reproducibility of the method. Based on this principle, DNPH assays showed aldehyde levels of 5.3% and 11.3% in the DAC 9% and 18% samples respectively. The precise determination of CHO contents was not possible for the 36% sample due to the variability in the UV measurement response. This result can be currently explained by the instability of the opened glucose ring in strongly oxidized DAC, especially in presence of primary amines as for DNPH. The non-linearity of Schiff base formation for DAC has already been demonstrated, lower levels of imine formation than the actual amount of CHO groups were observed [38]. On the other hand, the hydroxylamine hydrochloride reacted with aldehydes to form oximes and thus released hydrochloric acid (HCl) causing the pH drop of the medium. By titration of HCl with NaOH solution, it was possible to determine the molar number of aldehyde functions grafted on the surface of the polysaccharide backbone. Using this method, rates of 32.7%, 11.7% and 9% were obtained for DAC 36%, 18% and 9% respectively.

### 3.2. Strategy for Producing the DAC/PVA Scaffolds

**Figure 3:**
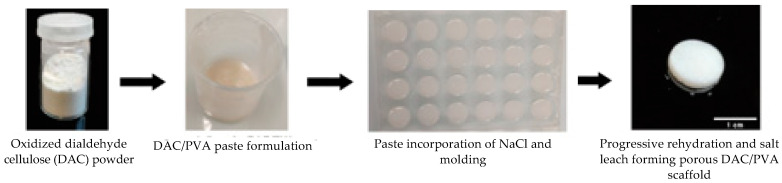
Photographs of the moulding process and formulation of scaffolds based on partial dialdehyde cellulose (here, DAC 9%/PVA (2:1) 0–500 µm NaCl).

The proposed protocol for the synthesis and shaping of cellulose/PVA scaffolds based on the functionalization of cellulose through aldehyde functions via classical sodium periodate oxidation was schematized in Figure 3. For all DAC/PVA formulations, the scaffold fabrication was allowed via a performant remodelling of both polymeric cellulose and PVA networks using a simple degeneration process in an NMP/LiCl system, followed by the regeneration of a dual robust composite in ethanol. A cohesive material can be obtained starting from a 9% dialdehyde functionalization and a DAC/PVA ratio of 1:2. Progressive heating of the polymer suspension led to complete dissolution of the powders between 30 and 50 °C, gelation of the PVA around 70 °C and finally to the formation of a smooth, viscous and homogeneous paste when the temperature of 90 °C was reached. The gradual reconstitution of the networks in ethanol generated a hard and rigid hydrogel that became flexible once fully hydrated in water.

**Figure 4:**
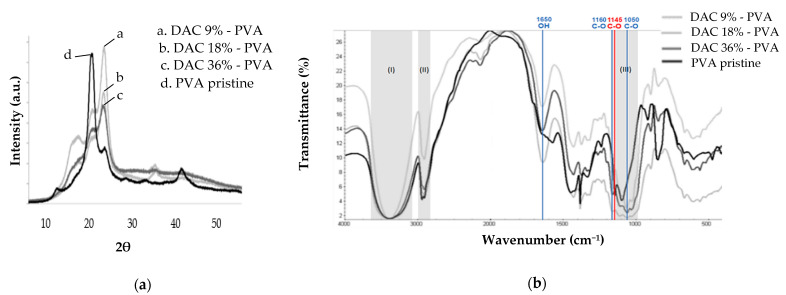
PVA and DAC-based composite scaffolds (**a**) XRD diffractograms; (**b**) FT-IR spectra.

In Figure 4, the XRD profiles of the cellulosic materials demonstrated a diffuse peak from 15° to 16° and 3 additional peaks at 20 °C, 22.5° and 40° respectively attributed to the (100), $\left(1\bar{1}0\right)$, (012), (200) and (004) crystallographic planes. This type of profile was identical to what is commonly observed for type I microcrystalline cellulose [39,40,41]. Moreover, these profiles differed greatly from the PVA pattern. The crystallinity of the scaffolds was thus essentially related to their microcrystalline cellulose composition (I = 68%) [42]. The crystallinity index of the cellulosic samples based on DAC 9%, 18% and 36% were, respectively, 62.5, 62.3 and 51.0%. The crystallinity of the samples decreased progressively by oxidation with sodium periodate, demonstrating aldehyde functions formation starting from the furanose ring opening essentially localized at cellulose backbone extremities. The peak at 40° disappeared in the case of DAC 36%, suggesting a slight modification of chains conformation. For DAC 9% and 18%, as all the signals were observed, long domains of the cellulose backbone were assumed to remain unchanged.

The FTIR spectra logically displayed signature signals for PVA and cellulose, both basic constituents of the materials. For DAC, a detailed description of the FT-IR spectrum can be found in the literature [43]. For PVA, at 3373 cm^−1^ the broadband (I) corresponds to hydroxyl OH groups involved in intermolecular and intramolecular hydrogen bonds. The 2918 cm^−1^ bands (II) and 1439 cm^−1^ bands represent CH polymer backbone stretching. The CH_2_ from the long alkyl chain vibrations were observed at 2052 cm^−1^. The 1728 cm^−1^ inflexion and 1097–1149 cm^−1^ multiple bands, in the fingerprint region (III), report C-O stretching. There, the 1145 cm^−1^ band is associated with PVA crystallinity nature (red) [44,45,46,47]. These peaks were found on the spectra of cellulosic materials, attesting to the maintenance of the PVA structure.

As cellulose shares groups in common with PVA, many bands were found to be in common (grey) especially in regions I, II and III. For better visualization of the fingerprint region, the FTIR spectra limited to region 1800 cm^−1^ to 700 cm^−1^ was provided in Supplementary Materials, Figure S1. Cellulose specific vibrations (blue) related to C-O and OH groups were, respectively, identified at 1057 cm^−1^ at 1650 cm^−1^ [48,49]. These predominant hydroxyl groups in cellulose tend to adsorb moisture. Bound water is therefore most likely responsible for this last peak [50,51]. Furthermore, the peaks at 1376 and 1021 cm^−1^ were attributed to CH2 bending and C-O-C stretching vibration representative of a furanose ring or glucose sub-unit in the polysaccharide backbone. The characteristic signals of aldehyde groups currently found that around 1730 cm^−1^ or 2700 cm^−1^ do not appear in FT-IR charts of the formed hydrogels [52]. The presence of C-O vibrations at 1057 cm^−1^ supported by those found at 1160 cm^−1^ as well as the band at OH 890 cm^−1^, respectively, were attributed to acetal and hemiacetal bonds [53].

### 3.3. Structural Characterization of the DAC/PVA Scaffolds

**Figure 5:**
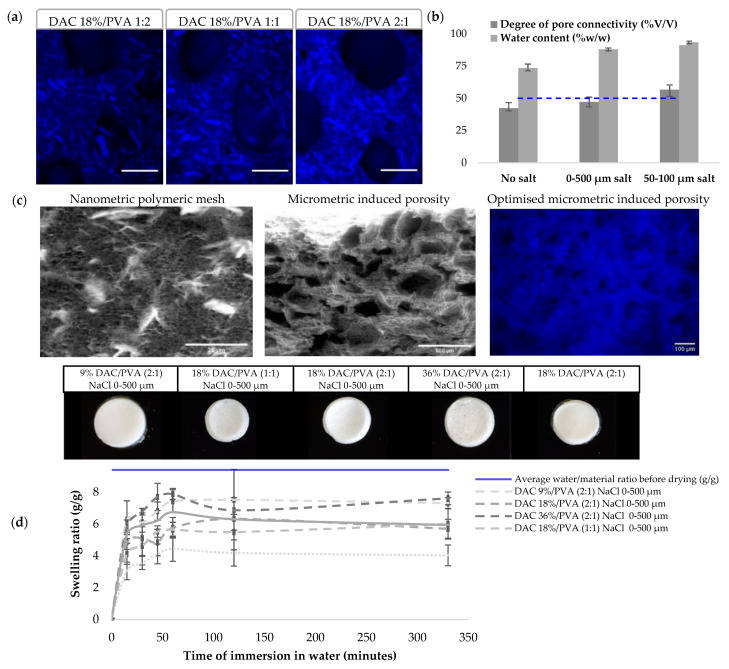
(**a**) Calcofluor white stained DAC 18%/PVA NaCl 0-500 µm scaffolds respectively at ratio 1:2, 1:1 and 2:1 showing cellulosic material (blue; scale bar: 250 µm); (**b**) Degree of pore connectivity (%*v*/*v*) and water content (%*w*/*w*) quantifications modulating salt leaching induced porosity (n = 5); (**c**) MEB observation of nano and micro porosities of DAC 18%/PVA (2:1) NaCl 0–500 µm scaffold; Calcofluor white stained DAC 9%/PVA (2:1) NaCl 50–100 µm showing enhanced porosity; (**d**) Swelling ratio study including photographs of rehydrated DAC/PVA based scaffolds.

Cellulose/PVA composite hydrogels were produced, controlling DAC degree of oxidation, DAC/PVA ratio and its porosity by the addition of porogen agents. Blue staining of the cellulose with calcofluor white stain allows for the observation of the macroscopic organisation of the polymeric network. In Figure 5, the increasing cellulose ratio can be interpreted as 50–150 µm long microcrystals homogeneously distributed in a hydrogel loaded with DAC fibres. This gel exhibits a micrometre-sized porosity attributable to its polymer network. Additional porosity was introduced through the solubilisation of NaCl salts of diameter calibrated between 0–500 µm or 50–100 µm demonstrating average diameters respectively of 305.4 ± 65.3 and 69.6 ± 16.2 µm. Homogeneous distribution of porosity was obtained, but the juxtaposition of the salts does not guarantee the interconnectivity of the pores required for biological applications. Indeed, the hydrogels present high-water contents (above 75% *w*/*w*) and an increasing porosity mediated by salt leaching. The degree of interconnectivity obtained by absorption of the free water contained in the hydrogel shows limited interconnectivity with the use of salts 0–500 µm. This was improved by the calibration of salts at 50–100 µm passing from 47.1 to 56.6%. To determine scaffolds potential applications in a biomedical context, we had to evaluate their swelling behaviour and mechanical properties. Dry materials showed similar rehydration profiles. After 1 h, a maximum swelling was reached. The hydrogel retained 4 to 8 g of water per g of dried hydrogel, representing 42.6 to 80.0% of the initial average water/dry mass ratio. The deformation of hydrogel was observed for all samples except for the 9% DAC/PVA (2:1) samples whose porosity was induced by salts of diameter 0–500 µm.

**Figure 6:**
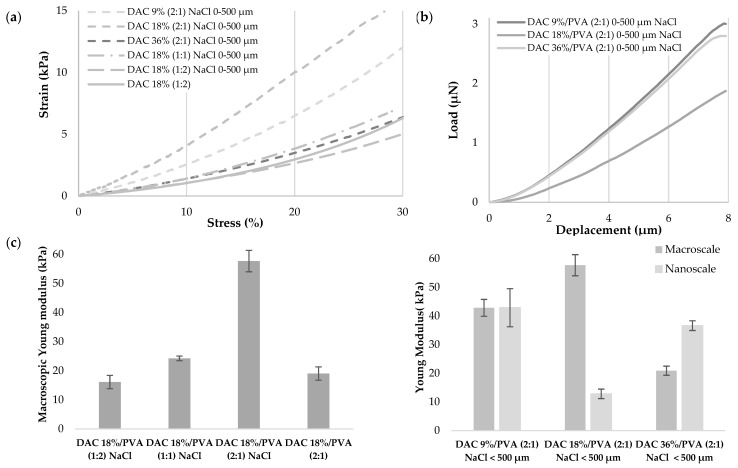
DAC/PVA hydrogels mechanical properties (**a**) Stress-strain curves obtained by compressive test. Compression up to 30%, speed: 0.01 mm/s, sensor: 22N (n = 5); (**b**) Load-deplacement curves obtained upon 15,000 nm at 3 nm/s nanoindentation. Indenter diameter: 28.5 μm, stiffness: 0.47 N/m (n = 3) (**c**) Determination of macroscopic and nanoscopic Young’s modulus. All conditions display *** significance except DAC 9%/PVA (2:1) NaCl < 500 µm samples (*** *p*-values < 0.001 (ANOVA, Tukey test)).

In Figure 6, the material mechanics were investigated through the estimation of Young’s modulus at macroscopic and nanoscopic scales respectively by compression and nanoindentation. Macroscopically, a gradual increase in DAC proportion significantly increased the Young’s modulus, as DAC 18%/PVA ratio of (1:2), (1:1) and (2:1) respectively depict Young’s moduli of 16.16, 24.25 and 57.21 kPa. The degree of oxidation of the cellulose dialdehyde also seemed to influence the mechanical properties of the hydrogels since Young’s moduli of 42.81, 57.69, 20.9 kPa are obtained by compression for hydrogels respectively based on 9%, 18% and 36% DAC. The introduction of salt-leached porosity significantly increased the stiffness of the material moving from a modulus of 19.05 to 57.21 kPa for a DAC 18%/PVA (2:1) hydrogels. At a nanometric level, when DAC 9% based materials demonstrated identical Young’s moduli compared to the macro metric level of 42.86 kPa, DAC 18% and 36% based materials showed lower moduli, respectively, of 12.83 and 36.63 kPa.

### 3.4. Investigation of DAC/PVA Scaffold Potential Use for Tissue Engineering, Implantable Matrices

**Figure 7:**
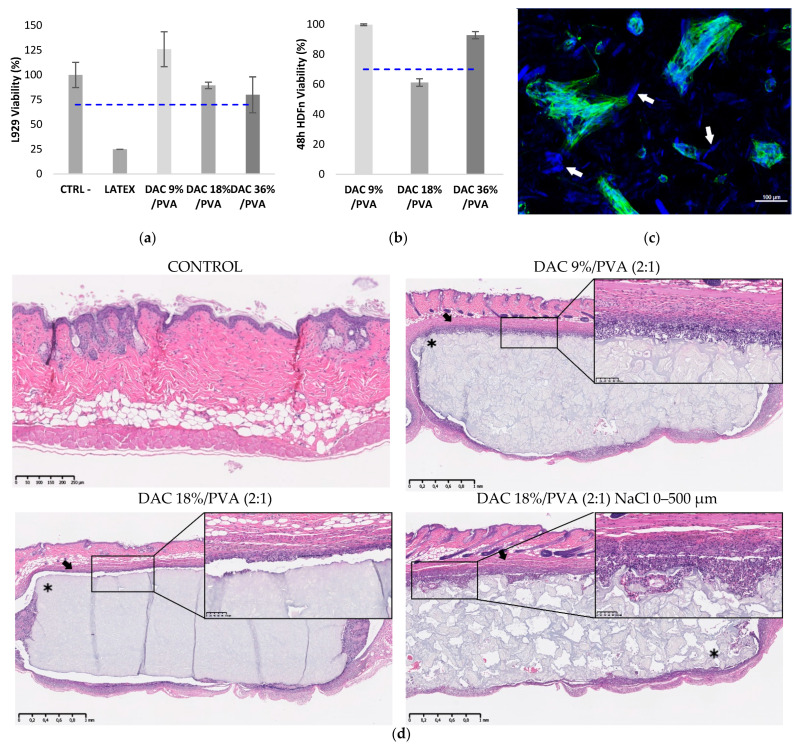
(**a**) DAC/PVA (2:1) NaCl hydrogels cytocompatibility test following the ISO10993 standard protocol (n = 3); (**b**) Neonatal human dermal fibroblast (HDFn) cultured on DAC/PVA (2:1) NaCl hydrogels viability assay at 48 h (n = 3); (**c**) Observation of adhered HDFn and spheroid-like structure on 9% DAC/PVA (2:1) material. DAPI: cell nucleus (blue); Phalloidin: cell cytoskeleton (green); White arrow: cellulose. (**d**) Examination of *in vivo* biocompatibility and biostability of nanoporous and macroporous cellulosic materials in a nude mouse model through histological study upon 1-month subcutaneous implantation (n = 3). *: cellulosic implant; arrow: fibrous capsule.

Firstly, indirect and direct cell viability were assessed in Figure 7 using regulatory reference cell lineage L929 and for its intended use, primary neonatal human dermal fibroblasts HDFn. Thus, L929 viabilities of 126.0, 89.4 and 79.9% were, respectively, obtained with 9%, 18%, 36% DAC-based hydrogels to a relatively untreated control considered at 100%. The cytotoxic latex used as positive control showed drop-in viability down to 24.9%, while HDF viabilities of 99.7% and 92.8% were achieved with the 9% and 36% DAC-based hydrogels. 

A low level of viability of 61.2% was reported for 18% oxidized cellulose-based hydrogel. HDF culture at the surface of porous materials showed good infiltration and pore occupation, with clusters of cells showing both a spread-out actin cytoskeleton that appears to anchor to nearby accessible cellulose particles (white arrows), as well as spheroid-like aggregations of cells.

One-month implantation of all materials in thymus-free nude mice showed no rejection of the implants, demonstrating their physical stability. As shown in Figure 7d, the material was completely recovered with no breakage, fragmentation or little to no sign of degradation for all porous and non-porous materials. The implanted area showed at the material interface a thin fibrous capsule presenting mild to moderate neutrophilic and macrophages inflammatory infiltrates. The DAC/PVA materials (2:1) of DAC 9%, 18%, 36% displayed mean inflammatory scores of 3, 2.5 and 2.5, respectively (n = 3). The addition of porosity increased the inflammation score to 3.3 for the DAC 18%/PVA (2:1) material.

## 4. Discussion

The loss of soft tissue, fatty, fibrous or muscular tissue affects a wide spectrum of patients, as it is associated with multiple medical conditions related to trauma, burns, infections, tumour removals, degenerative diseases or autoimmune disorders. In addition to the physical impact of their tissue loss, patients encounter psychological cues linked to the loss of normal body contours that only can be remedied by filling in the affected area. Although common, autologous fat injection presents a need for repeated intervention questioning resulting viability and short term stability [54,55]. Practitioners are still seeking an adequate methodology that can sustain stable volume and long-term tissue regeneration. Thus, biocompatible filling hydrogels such as our DAC/PVA substitute are particularly promising for the filling of small to large tissue defects. 

Our synthesis methodology allowed us to obtain a ready-to-use paste that can be declined in multiple forms, membrane, scaffold or beads completed by moulding, additive manufacturing, extrusion or electrospinning mainly for biomedical applications. Regarding DAC/PVA hydrogel synthesis, effective functionalisation of cellulose was obtained using the classical periodate sodium oxidation method. While FTIR and XDR proved the absence of aldehyde groups in our final cellulosic materials, those analyses provide insight into their formation mechanism, high reactivity and resulting bonding. Thus, FT-IR analysis highlighted the cellulose and PVA composition of the DAC/PVA materials but did not distinguish the dialdehyde functions distinctly. Subsequent signals attributed to acetal and hemiacetal bonds were identified, demonstrating the effectiveness of the NMP/LiCl system for simultaneously strongly cross-link PVA and DAC and engage electrostatic interactions to stabilise the hydrogels. XDR demonstrated a significant decrease of the crystallinity index linked to the cellulose increasing degree of oxidation [56]. This loss of crystallinity was considered to result from the opening of glucopyranose rings and the destruction of their ordered packing. This occurs mainly at the extremities of the accessible polymeric chains generating a fragmentation of the particles by disruption of the interchain hydrogen bonds [57]. As mentioned in the literature, the number average molecular weight (M_n_) of biopolymers tends to be reduced as a result of oxidation [58]. Given the indirect evidence of aldehyde groups, further quantifications were undertaken with DNPH and hydroxylamine hydrochloride to characterise our DAC powders. These methods have been widely used and prove to be very sensitive and accurate for the quantification of aldehyde content of polysaccharides [57,59]. Lower rates of respectively 5–9%, 11–12% and 33% were thus obtained for the theoretically 9%, 18% and 36% DAC powders. Cellulose and DAC powders are difficult to solubilise. Indeed, DAC is hardly soluble in water, even when heated, especially on dry or less than 75% oxidized products [60]. The aldehyde functionalities are present in a complex interconverting mixture of hydrates, intramolecular and intermolecular hemiacetals and acetals formed during reaction and drying and are therefore not in their free form to react [59,61]. DAC quantifications may be slightly underestimated by lack of accessibility and difficult reversibility of established bonds in water. Moreover, under our heating conditions in an acidic medium (post-reaction pH = 4) and especially in the presence of high concentrations of sodium periodate (36% mol/mol of cellulose), we observed significant hydrolysis of our product visualized through a very significant decrease in yield. Indeed, periodate oxidation is an incomplete reaction whose rate and yield depend on temperature, pH, reaction time and oxidant concentration. Higher temperature tends to produce better oxidation rates but lower yield, degrading both oxidant and DAC through acid catalysed cleavage of the β-(1→4) glycosidic bonds, fragmenting it in a heterogeneous manner [56]. The optimisation of sodium periodate oxidation relies on minimising the time and temperature of oxidation to limit cellulose hydrolysis. The addition of salts such as LiCl proves to be effective for that purpose in reducing inter and intrachain interactions, but LiCl-catalysed periodate degradation has also been observed [62]. During our studies, microwave heating has been considered and tested as a substitute for conventional heating for the reshaping and arrangement of cellulose and PVA polymers. However, these devices are limited in terms of production scale, agitation quality and reaction observability. Moreover, the conventional heating reaction time has been reduced to approach the one used in microwave heating. For our application, this method has thus lost all interest.

Cellulose/PVA composite hydrogels can be synthesized controlling DAC degree of oxidation, DAC/PVA ratio and its porosity by the addition of pore-forming agents. The physical characterization of our hydrogels reveals the homogeneity of the interconnected micrometric network and the incorporation of microcrystalline cellulose fibres, as well as the good integration of an additional porosity required for some biomedical applications. To reach sufficient interconnectivity of our additional porosity, a reduced granulometry of the salts was used to increase the surface area developed by these salts, thus favouring their juxtaposition and connection. Additional freezing-thawing was attempted to further connect the pores but the materials systematically returned to their initial shape, being very elastic. Their water absorption capacity was assessed to consider possible storage in a dry state and rehydration for use. However, only the 9% (2:1) NaCl 0–500 µm DAC samples were able to regain a sufficiently high-water content and shape to be considered. Indeed, higher oxidation rates of 18% and 36% develop more cross-links and electrostatic interactions which are not fully recovered when broken by drying. In addition, hydrogels with large cavities hold more free water and thus rehydrate more efficiently. By modulation of the DAC/PVA ratio, we find that cellulose stiffens the hydrogel at the macro metric level, most likely through the crystalline micro cellulose fibres previously observed. Moreover, salts dissolution must generate irreversible dehydration of the material in the pore area, explaining the observed stiffening of porous hydrogels. The degree of oxidation of cellulose being largely related to the size of the particles, an impact on the mechanics of the hydrogel at the macroscopic level is observed when it varies. We assume that the higher stiffness observed for the 9% and 18% samples are certainly related to the presence of larger microcrystalline cellulose particles compared to the 36% DAC particles which underwent more hydrolysis and fragmentation during oxidation. At the nanometric level, the 9% and 36% DAC-based materials show a similar Young’s modulus of about 40 kPa, while at 18% a significantly lower Young’s modulus is obtained. These observations are most certainly linked to the organisation of the DAC/PVA network, as well as the nature and density of the associated interactions and bonds. Our hydrogels have mechanical properties similar to those of soft tissues whose Young’s moduli range from 0.1 kPa to 1 MPa. By cellularisation, these matrices tend to stiffen as previously observed by deposition, remodelling and crosslinking of extracellular matrix protein notably collagen [63].

To consider any biomedical application, a rigorous study of the biological acceptability of this material has been undertaken. The release media of DAC/PVA hydrogels show decreasing viability of L929 as the degree of oxidation of DAC increases up to 18% DAC oxidation, in conformity with the ISO 10993-5 standard. Since PVA is known to be fully biocompatible, the indirect cytotoxicity observed on L929 is due to the release of cellulose dialdehyde particles. This same effect has already been observed on the NIH/3T3 fibroblast line where cell viability drops to 70% for 1.74 mmol/10 g PVA of CHO groups [64]. Despite being weakly oxidised, DAC predominates in the composition of our hydrogel, reaching a minimum of 23.38 mmol/10 g PVA at 9%. The absence of toxicity observed below 18% reflects the full engagement of aldehyde groups in stable linkages non-deleterious at the cellular level. When seeded on the material surface, the cell-DAC/PVA interaction shows a correlation between the cell viability of HDFs and the mechanical properties of the material at the nanometre level. Indeed, HDFs exhibit stronger cell viability for DAC 9% and DAC 36% then DAC 18% material respectively showing Young Modulus of around 37, 13 and 43 kPa. Although little data is available on DAC/PVA hydrogels, numerous studies show the positive impact in 2D and 3D of matrix stiffness on fibroblast activity in terms of cell adhesion, proliferation and migration [65,66]. The DAC 9% /PVA (2:1) matrix appears to be the most suitable material for *in vitro* use.

Given that the DAC/PVA materials have substantially identical microenvironments in their composition, very similar results are subsequently obtained for DAC 9%, 18% and 36% material during long-term cultivation. As homogeneous and total infiltration of the porous material is achieved, the induction of porosity with controlled salt granularity proves to be effective and sufficient in our case. When cultured for 3 weeks, seeded fibroblasts organise themselves into spheroid-like cell aggregates. Indeed, as a biologically inert polymer, PVA shows an inability to engage interactions with ECM proteins such as fibronectin, collagen, laminin and thus associate with cellular integrin receptors. The incorporation of microcrystalline cellulose in our hydrogel offers anchorage points for these cellular aggregates. Indeed, on cellulose surfaces, HDFs can establish cell adhesions and develop filopodia as the numerous hydroxyl groups on the cellulose surface allow the protein adsorption required for cell adhesion by electrostatic interactions and hydrogen bonds [67,68,69]. By chemical functionalization, oxidation, phosphorylation or specific chemical grafting, these interactions can be promoted to remedy growth factor or FBS supplementation [70]. Mechanically confined fibroblast has shown the ability to reprogram in stem-cell-like cells and rejuvenate [71,72]. In the literature, PVA and bacterial cellulose also demonstrated spheroid formation and long-term stem-cell maintenance properties [29,73,74]. The potential of these spheroid-like structures at the surface of our DAC/PVA material is very high, providing an environment recapitulating the in-vivo complexity better than the traditional 2D or some 3D cultures. These cells functionality is more representative for pharmacological model predictivity and exhibit promising regeneration properties for tissue engineering and regenerative medicine applications. 

Implanted DAC/PVA hydrogels exhibit complete stability by a month of implantation, a mild inflammation characterised by extensive neutrophil and macrophage infiltration and the formation of a thin fibrous capsule at the periphery of the implant. By inducing porosity in the implant, the contact surface is increased and cellular infiltration is facilitated, apparently increasing the inflammatory score. Such inflammation is indicative of a foreign body reaction (FBR), a key reaction in evaluating the safety of a scaffold in tissue engineering applications. Indeed, once implanted, our material interacts with its surroundings, through fluid, protein and cellular infiltration. Consisting of cellulose and PVA, the implant was recognised as non-self, and the body undertakes inflammatory processes aimed at its destruction or isolation from the rest of the body [75]. In the case of cellulose-based hydrogels, mild inflammation was also observed at 4 weeks and then disappeared at 8 weeks as the fibrous capsule surrounding the implant was refined [76,77]. Lacking a cellulase equivalent to breaking β 1-4 glycosidic bond *in vivo*, cellulose demonstrates slow in situ degradation via particle macrophage phagocytosis. Cellulosic materials can persist in the crystalline form up to 5 months after implantation [78]. To overcome such *vivo* stability and to reinforce cellulose fibres usage as a biomaterial for tissue engineering, the degradation of cellulose may bee modulated by incorporating cellulase into the material [79].

## 5. Conclusions

We have developed and characterised a material based on cellulose dialdehyde and PVA, which has shown soft tissue-like mechanical properties, cytocompatibility and the promising ability to form spheroid-like structures for the development of pharmacological models. Investigations should be conducted on different cell types to confirm and characterise these structures in terms of size, morphology, cell viability, metabolic activity and dedifferentiation/differentiation potential. The stability of this material *in vivo* also makes it a candidate for the development of raw, biochemically functionalized or cellularized implantable matrices. The synthesis path of our material also offers the possibility to produce innovative nanoscale culture substitutes for tissue engineering using nanocrystalline cellulose.

## Figures and Tables

**Table 1:** DNPH/Hydroxylamine CHO dosage (n = 3).

Theoretical DAC Levels (%)	Experimental DAC Levels Obtained via DNPH Method (%)	Experimental DAC Levels Obtained via Hydroxylamine Method (%)
9	5.3 ± 0.6	9.0 ± 1.0
18	11.3 ± 0.6	11.7 ± 1.5
36	ND	32.7 ± 0.6

## Data Availability

Data Availability Statement Raw data are stored in-house at UTC and can be made available upon request.

## Supplementary Materials

The following are available online at https://www.mdpi.com/article/10.3390/polym13203598/s1, Figure S1: Zoom of PVA and DAC-based composite scaffolds FT-IR spectra.

## References

[1] Zhu J., Marchant R.E. (2011). Design properties of hydrogel tissue-engineering scaffolds. Expert Rev. Med. Devices.

[2] Fan C., Wang D.-A. (2017). Macroporous Hydrogel Scaffolds for Three-Dimensional Cell Culture and Tissue Engineering. Tissue Eng. Part B Rev..

[3] Annabi N., Nichol J.W., Zhong X., Ji C., Koshy S., Khademhosseini A., Dehghani F. (2010). Controlling the Porosity and Microarchitecture of Hydrogels for Tissue Engineering. Tissue Eng. Part B Rev..

[4] Troy E., Tilbury M.A., Power A.M., Wall J.G. (2021). Nature-Based Biomaterials and Their Application in Biomedicine. Polymers.

[5] Chaudhari A., Vig K., Baganizi D., Sahu R., Dixit S., Dennis V., Singh S., Pillai S. (2016). Future Prospects for Scaffolding Methods and Biomaterials in Skin Tissue Engineering: A Review. Int. J. Mol. Sci..

[6] Gsib O., Duval J.-L., Goczkowski M., Deneufchatel M., Fichet O., Larreta-Garde V., Bencherif S., Egles C. (2017). Evaluation of Fibrin-Based Interpenetrating Polymer Networks as Potential Biomaterials for Tissue Engineering. Nanomaterials.

[7] Dutta S.D., Patel D.K., Lim K.-T. (2019). Functional cellulose-based hydrogels as extracellular matrices for tissue engineering. J. Biol. Eng..

[8] Kale R.D., Bansal P.S., Gorade V.G. (2018). Extraction of Microcrystalline Cellulose from Cotton Sliver and Its Comparison with Commercial Microcrystalline Cellulose. J. Polym. Environ..

[9] Ludueña L.N., Vecchio A., Stefani P.M., Alvarez V.A. (2013). Extraction of cellulose nanowhiskers from natural fibers and agricultural byproducts. Fibers Polym..

[10] Rosa S.M.L., Rehman N., de Miranda M.I.G., Nachtigall S.M.B., Bica C.I.D. (2012). Chlorine-free extraction of cellulose from rice husk and whisker isolation. Carbohydr. Polym..

[11] Pirani S., Hashaikeh R. (2013). Nanocrystalline cellulose extraction process and utilization of the byproduct for biofuels production. Carbohydr. Polym..

[12] Danial W.H., Abdul Majid Z., Mohd Muhid M.N., Triwahyono S., Bakar M.B., Ramli Z. (2015). The reuse of wastepaper for the extraction of cellulose nanocrystals. Carbohydr. Polym..

[13] Bergenstråhle M., Wohlert J., Himmel M.E., Brady J.W. (2010). Simulation studies of the insolubility of cellulose. Carbohydr. Res..

[14] Zhang C., Liu R., Xiang J., Kang H., Liu Z., Huang Y. (2014). Dissolution Mechanism of Cellulose in N, N-Dimethylacetamide/Lithium Chloride: Revisiting through Molecular Interactions. J. Phys. Chem. B.

[15] Peng H., Wang S., Xu H., Hao X. (2017). Preparation, properties and formation mechanism of cellulose/polyvinyl alcohol bio-composite hydrogel membranes. New J. Chem..

[16] Maharjan B., Park J., Kaliannagounder V.K., Awasthi G.P., Joshi M.K., Park C.H., Kim C.S. (2021). Regenerated cellulose nanofiber reinforced chitosan hydrogel scaffolds for bone tissue engineering. Carbohydr. Polym..

[17] Krüger M., Oosterhoff L.A., van Wolferen M.E., Schiele S.A., Walther A., Geijsen N., De Laporte L., van der Laan L.J.W., Kock L.M., Spee B. (2020). Cellulose Nanofibril Hydrogel Promotes Hepatic Differentiation of Human Liver Organoids. Adv. Healthc. Mater..

[18] Kumar A., Lee Y., Kim D., Rao K.M., Kim J., Park S., Haider A., Lee D.H., Han S.S. (2017). Effect of crosslinking functionality on microstructure, mechanical properties, and in vitro cytocompatibility of cellulose nanocrystals reinforced poly (vinyl alcohol)/sodium alginate hybrid scaffolds. Int. J. Biol. Macromol..

[19] Du H., Liu W., Zhang M., Si C., Zhang X., Li B. (2019). Cellulose nanocrystals and cellulose nanofibrils based hydrogels for biomedical applications. Carbohydr. Polym..

[20] Kumar A., Matari I.A.I., Han S.S. (2020). 3D printable carboxylated cellulose nanocrystal-reinforced hydrogel inks for tissue engineering. Biofabrication.

[21] Jayaramudu T., Ko H.-U., Zhai L., Li Y., Kim J. (2017). Preparation and characterization of hydrogels from polyvinyl alcohol and cellulose and their electroactive behavior. Soft Mater..

[22] Jayaramudu T., Hyun-U K., Xiaoyuan G., Yaguang L., Sang Youn K., Jaehwan K., Varadan V.K. (2016). Cellulose/Polyvinyl Alcohol-Based Hydrogels for Reconfigurable Lens.

[23] Zhang M., Dong X., Ouyang Y., Li Y., Liu T., Cheng T., Minli H., Ma J., Li G. (2016). Vapor-Phase Oxidation of Ethylene to Produce Vinyl Acetate. Chinese Patent.

[24] Liu Y., Hu H., Yang X., Lv J., Zhou L., Luo Z. (2019). Hydrophilic modification on polyvinyl alcohol membrane by hyaluronic acid. Biomed. Mater..

[25] Cooper T.A., Farmer N. (2013). 5-Developments in bioplastic materials for packaging food, beverages and other fast-moving consumer goods. Trends in Packaging of Food, Beverages and Other Fast-Moving Consumer Goods (FMCG).

[26] Gupta S., Goswami S., Sinha A. (2012). A combined effect of freeze--thaw cycles and polymer concentration on the structure and mechanical properties of transparent PVA gels. Biomed. Mater..

[27] Schmedlen R.H., Masters K.S., West J.L. (2002). Photocrosslinkable polyvinyl alcohol hydrogels that can be modified with cell adhesion peptides for use in tissue engineering. Biomaterials.

[28] Zajaczkowski M.B., Cukierman E., Galbraith C.G., Yamada K.M. (2003). Cell–Matrix Adhesions on Poly(vinyl alcohol) Hydrogels. Tissue Eng..

[29] Molyneaux K., Wnek M.D., Craig S.E.L., Vincent J., Rucker I., Wnek G.E., Brady-Kalnay S.M. (2021). Physically-cross-linked poly(vinyl alcohol) cell culture plate coatings facilitate preservation of cell-cell interactions, spheroid formation, and stemness. J. Biomed. Mater. Res. B Appl. Biomater..

[30] Hsieh H.-Y., Young T.-H., Yao C.-C., Chen Y.-J. (2019). Aggregation of human dental pulp cells into 3D spheroids enhances their migration ability after reseeding. J. Cell. Physiol..

[31] Huang Y.-C., Chan C.-C., Lin W.-T., Chiu H.-Y., Tsai R.-Y., Tsai T.-H., Chan J.-Y., Lin S.-J. (2013). Scalable production of controllable dermal papilla spheroids on PVA surfaces and the effects of spheroid size on hair follicle regeneration. Biomaterials.

[32] Nuttelman C.R., Mortisen D.J., Henry S.M., Anseth K.S. (2001). Attachment of fibronectin to poly(vinyl alcohol) hydrogels promotes NIH3T3 cell adhesion, proliferation, and migration. J. Biomed. Mater. Res..

[33] Peng L., Zhou Y., Lu W., Zhu W., Li Y., Chen K., Zhang G., Xu J., Deng Z., Wang D. (2019). Characterization of a novel polyvinyl alcohol/chitosan porous hydrogel combined with bone marrow mesenchymal stem cells and its application in articular cartilage repair. BMC Musculoskelet. Disord..

[34] Pan Z., Yin H., Wang S., Xiong G., Yin Z. (2021). Potential In Vitro Tissue-Engineered Anterior Cruciate Ligament by Copolymerization of Polyvinyl Alcohol and Collagen. J. Craniofac. Surg..

[35] Rasband W.S., Eliceiri K.W. (1997). Image J.

[36] Zhao H., Heindel N.D. (1991). Determination of Degree of Substitution of Formyl Groups in Polyaldehyde Dextran by the Hydroxylamine Hydrochloride Method. Pharm. Res..

[37] Segal L., Creely J.J., Martin A.E., Conrad C.M. (1959). An Empirical Method for Estimating the Degree of Crystallinity of Native Cellulose Using the X-Ray Diffractometer. Text. Res. J..

[38] Leguy J. (2018). Periodate Oxidation of Cellulose for Internal Plasticization and Materials Design.

[39] Melo A.R.A., Filho J.C.D., Neto R.P.C., Ferreira W.S., Archanjo B.S., Curti R.V., Tavares M.I.B. (2020). Effect of Ultra-Turrax on Nanocellulose Produced by Acid Hydrolysis and Modified by Nano ZnO by Sol-Gel Method. Mater. Sci. Appl..

[40] Wei J., Du C., Liu H., Chen Y., Yu H., Zhou Z. (2016). Preparation and Characterization of Aldehyde-Functionalized Cellulosic Fibers through Periodate Oxidization of Bamboo Pulp. BioResources.

[41] Mihranyan A., Llagostera A.P., Karmhag R., Strømme M., Ek R. (2004). Moisture sorption by cellulose powders of varying crystallinity. Int. J. Pharm..

[42] Dang X., Liu P., Yang M., Deng H., Shan Z., Zhen W. (2019). Production and characterization of dialdehyde cellulose through green and sustainable approach. Cellulose.

[43] Barghamadi M., Barghamadi G., Raouf M., Raouf A. (2009). Non-isothermal Cure Kinetics of Diglycidyl Ether of Bisphenol-A with Various Aromatic Diamines. Iran. Polym. J..

[44] Suganthi S., Vignesh S., Kalyana Sundar J., Raj V. (2020). Fabrication of PVA polymer films with improved antibacterial activity by fine-tuning via organic acids for food packaging applications. Appl. Water Sci..

[45] Siddaiah T., Ojha P., Kumar N.O.G.V.R., Ramu C. (2018). Structural, Optical and Thermal Characterizations of PVA/MAA:EA Polyblend Films. Mater. Res..

[46] dos Reis E.F., Campos F.S., Lage A.P., Leite R.C., Heneine L.G., Vasconcelos W.L., Lobato Z.I.P., Mansur H.S. (2006). Synthesis and characterization of poly (vinyl alcohol) hydrogels and hybrids for rMPB70 protein adsorption. Mater. Res..

[47] Mansur H.S., Oréfice R.L., Mansur A.A.P. (2004). Characterization of poly(vinyl alcohol)/poly(ethylene glycol) hydrogels and PVA-derived hybrids by small-angle X-ray scattering and FTIR spectroscopy. Polymer.

[48] Togrul H. (2003). Flow properties of sugar beet pulp cellulose and intrinsic viscosity–molecular weight relationship. Carbohydr. Polym..

[49] Jia N., Li S.-M., Ma M.-G., Zhu J. (2011). Synthesis and characterization of cellulose-silica composite fiber in ethanol/water mixed solvents. BioResources.

[50] Lv P., Almeida G., Perré P. (2015). TGA-FTIR Analysis of Torrefaction of Lignocellulosic Components (cellulose, xylan, lignin) in Isothermal Conditions over a Wide Range of Time Durations. BioResources.

[51] Zhuang J., Li M., Pu Y., Ragauskas A., Yoo C. (2020). Observation of Potential Contaminants in Processed Biomass Using Fourier Transform Infrared Spectroscopy. Appl. Sci..

[52] Nandiyanto A.B.D., Oktiani R., Ragadhita R. (2019). How to Read and Interpret FTIR Spectroscope of Organic Material. Indones. J. Sci. Technol..

[53] da Silva A.R.P., Macedo T.L., Coletta D.J., Feldman S., Pereira M.d.M. (2016). Synthesis, characterization and cytotoxicity of Chitosan/Polyvinyl Alcohol/Bioactive Glass hybrid scaffolds obtained by lyophilization. Matér. Rio Jan..

[54] Delay E., Guerid S., Meruta A.C. (2018). Indications and Controversies in Lipofilling for Partial Breast Reconstruction. Clin. Plast. Surg..

[55] Simonacci F., Bertozzi N., Grieco M.P., Grignaffini E., Raposio E. (2017). Procedure, applications, and outcomes of autologous fat grafting. Ann. Med. Surg..

[56] Li H., Wu B., Mu C., Lin W. (2011). Concomitant degradation in periodate oxidation of carboxymethyl cellulose. Carbohydr. Polym..

[57] Kim U.-J., Kuga S., Wada M., Okano T., Kondo T. (2000). Periodate Oxidation of Crystalline Cellulose. Biomacromolecules.

[58] Plappert S.F., Quraishi S., Pircher N., Mikkonen K.S., Veigel S., Klinger K.M., Potthast A., Rosenau T., Liebner F.W. (2018). Transparent, Flexible, and Strong 2,3-Dialdehyde Cellulose Films with High Oxygen Barrier Properties. Biomacromolecules.

[59] Tummalapalli M., Gupta B. (2015). A UV-Vis Spectrophotometric Method for the Estimation of Aldehyde Groups in Periodate-Oxidized Polysaccharides Using *2,4*-Dinitrophenyl Hydrazine. J. Carbohydr. Chem..

[60] Kim U.-J., Wada M., Kuga S. (2004). Solubilization of dialdehyde cellulose by hot water. Carbohydr. Polym..

[61] Siller M., Amer H., Bacher M., Roggenstein W., Rosenau T., Potthast A. (2015). Effects of periodate oxidation on cellulose polymorphs. Cellulose.

[62] Sirvio J., Hyvakko U., Liimatainen H., Niinimaki J., Hormi O. (2011). Periodate oxidation of cellulose at elevated temperatures using metal salts as cellulose activators. Carbohydr. Polym..

[63] Tupin S., Molimard J., Cenizo V., Hoc T., Sohm B., Zahouani H. (2016). Multiscale Approach to Characterize Mechanical Properties of Tissue Engineered Skin. Ann. Biomed. Eng..

[64] Münster L., Capáková Z., Fišera M., Kuřitka I., Vícha J. (2019). Biocompatible dialdehyde cellulose/poly(vinyl alcohol) hydrogels with tunable properties. Carbohydr. Polym..

[65] Asano S., Ito S., Takahashi K., Furuya K., Kondo M., Sokabe M., Hasegawa Y. (2017). Matrix stiffness regulates migration of human lung fibroblasts. Physiol. Rep..

[66] Hadjipanayi E., Mudera V., Brown R.A. (2009). Close dependence of fibroblast proliferation on collagen scaffold matrix stiffness. J. Tissue Eng. Regen. Med..

[67] Khan S., Ul-Islam M., Ullah M.W., Ikram M., Subhan F., Kim Y., Jang J.H., Yoon S., Park J.K. (2015). Engineered regenerated bacterial cellulose scaffolds for application in in vitro tissue regeneration. RSC Adv..

[68] Mohammadi F., Moeeni M., Li C., Boukherroub R., Szunerits S. (2020). Interaction of cellulose and nitrodopamine coated superparamagnetic iron oxide nanoparticles with alpha-lactalbumin. RSC Adv..

[69] Orelma H., Filpponen I., Johansson L.-S., Laine J., Rojas O.J. (2011). Modification of Cellulose Films by Adsorption of CMC and Chitosan for Controlled Attachment of Biomolecules. Biomacromolecules.

[70] Courtenay J., Sharma R., Scott J. (2018). Recent Advances in Modified Cellulose for Tissue Culture Applications. Molecules.

[71] Roy B., Venkatachalapathy S., Ratna P., Wang Y., Jokhun D.S., Nagarajan M., Shivashankar G.V. (2018). Laterally confined growth of cells induces nuclear reprogramming in the absence of exogenous biochemical factors. Proc. Natl. Acad. Sci. USA.

[72] Roy B., Yuan L., Lee Y., Bharti A., Mitra A., Shivashankar G.V. (2020). Fibroblast rejuvenation by mechanical reprogramming and redifferentiation. Proc. Natl. Acad. Sci. USA.

[73] Tronser T., Laromaine A., Roig A., Levkin P.A. (2018). Bacterial Cellulose Promotes Long-Term Stemness of mESC. ACS Appl. Mater. Interfaces.

[74] Okita Y., Zheng L., Kawanishi K., Miyoshi H., Yanagihara K., Kato M. (2021). Polyvinyl alcohol scaffolds and supplementation support 3D and sphere culturing of human cancer cell lines by reducing apoptosis and promoting cellular proliferation. Genes Cells.

[75] Klopfleisch R., Jung F. (2017). The pathology of the foreign body reaction against biomaterials: Foreign Body Reaction to Biomaterials. J. Biomed. Mater. Res. A.

[76] Ramphul H., Gimié F., Andries J., Jhurry D., Bhaw-Luximon A. (2020). Sugar-cane bagasse cellulose-based scaffolds promote multi-cellular interactions, angiogenesis and reduce inflammation for skin tissue regeneration. Int. J. Biol. Macromol..

[77] Modulevsky D.J., Cuerrier C.M., Pelling A.E. (2016). Biocompatibility of Subcutaneously Implanted Plant-Derived Cellulose Biomaterials. PLoS ONE.

[78] Singh M., Ray A.R., Verma P.V.K., Guha S.K. (1979). Potential Biosoluble Carriers: Biocompatibility and Biodegradability of Oxidized Cellulose. Biomater. Med. Devices Artif. Organs.

[79] Kamdem Tamo A., Doench I., Morales Helguera A., Hoenders D., Walther A., Madrazo A.O. (2020). Biodegradation of Crystalline Cellulose Nanofibers by Means of Enzyme Immobilized-Alginate Beads and Microparticles. Polymers.

