# Supplementary figures and images for: Cellulosic/Polyvinyl Alcohol Composite Hydrogel: Synthesis, Characterization and Applications in Tissue Engineering
Source: Polymers (Basel). 2021 Oct 19;13(20):3598. doi: 10.3390/polym13203598 (PMC8539384; doi:10.3390/polym13203598)

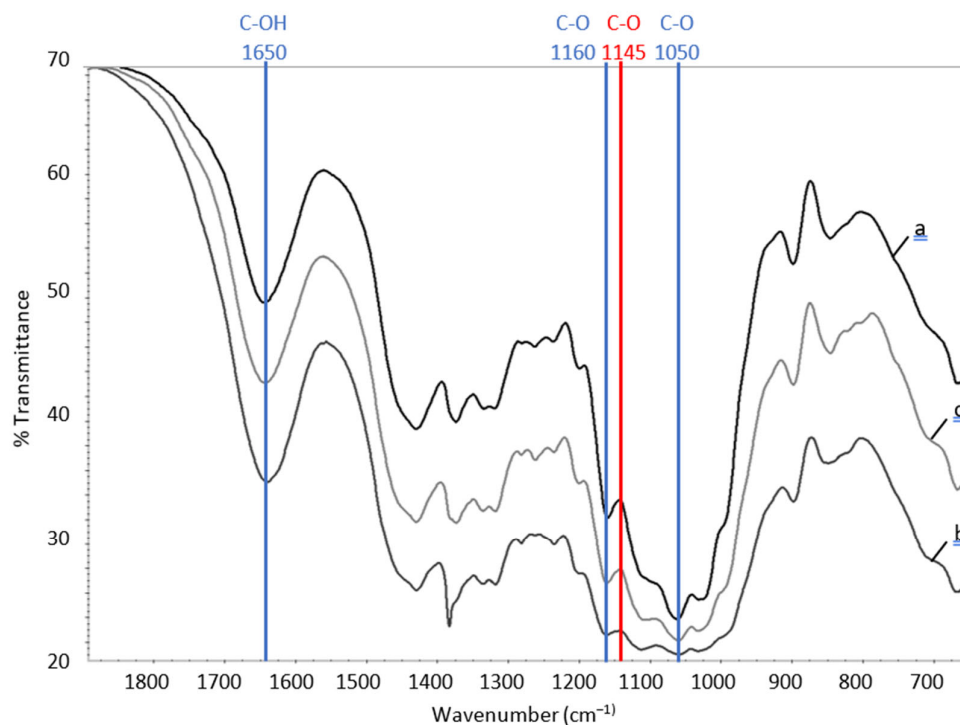

**Figure S1.** Zoom of PVA and DAC-based composite scaffolds FT-IR spectra (a: DAC 9%, b: DAC 18%, c: DAC 36%/PVA (2:1) NaCl)

Supplement: Supplementary file 1 [file polymers-13-03598-s001.zip › polymers-1411614-supplementary.pdf]
